# Medical Management of Heyde Syndrome

**DOI:** 10.7759/cureus.12551

**Published:** 2021-01-07

**Authors:** Samridhi Sinha, Daniel Castro, Shams Shakil

**Affiliations:** 1 Internal Medicine, The Brooklyn Hospital Center/Mount Sinai Heart, Brooklyn, USA; 2 Hemotolgy and Oncology, The Brooklyn Hospital Center/Mount Sinai Heart, Brooklyn, USA; 3 Internal Medicine, The Brooklyn Hospital Center, Brooklyn, USA; 4 Hematology and Oncology, The Brooklyn Hospital Center/Mount Sinai Heart, Brooklyn, USA

**Keywords:** heyde syndrome, anemia, aortic stenosis, colonic angiodysplasia, acquired coagulopathy, arteriovenous malformations, acquired vwf factor deficiency

## Abstract

Heyde syndrome is a triad of bleeding colonic angiodysplasia, aortic stenosis, and acquired coagulopathy. It is most commonly seen in the elderly between 60-80 years of age. We present a case of Heyde syndrome presenting with severe anemia secondary to bleeding angiodysplasia or arteriovenous malformations (AVM) in the lower gastrointestinal (GI) tract.

## Introduction

Heyde syndrome is an acquired multi-organ disorder characterized by a triad of anemia due to bleeding colonic angiodysplasia, aortic stenosis, and acquired coagulopathy. In 1958, E.C. Heyde described the Heyde syndrome in his letter to the *New England Journal of Medicine* based on his observation that elderly patients with aortic stenosis are at an increased risk of gastrointestinal bleeding [1].^ ^

## Case presentation

**Figure 1:**
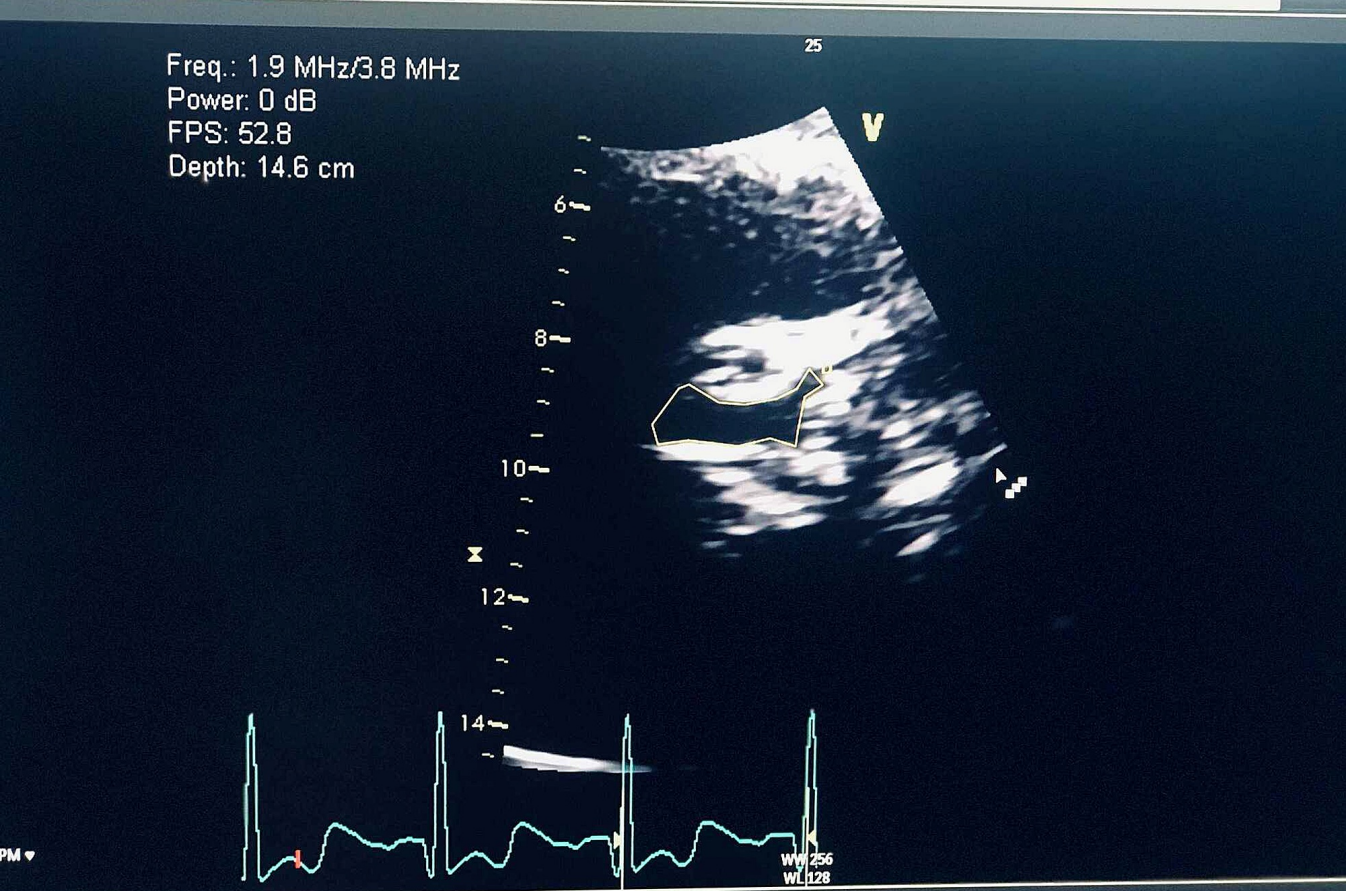
Transthoracic echocardiogram showing moderate calcification of the aortic leaflets Area demarcated by yellow line shows aortic stenosis.

An 85-year-old female with a past medical history of lower gastrointestinal bleeding secondary to arteriovenous malformations status post-hemicolectomy, hypertension, non-obstructive coronary artery disease, hypertrophic obstructive cardiomyopathy status postablation was admitted for symptomatic anemia with complaints of melena, dizziness, and shortness of breath for two to three weeks. The patient denied chest pain; hemoglobin on admission was 5 g/dl (normal range 11.5-14.1 g/dl in female population); fecal occult blood was positive at the time of admission. The patient's vitals were stable. After receiving several units of blood during admission, hemoglobin improved to 9.3 g/dl. Colonoscopy was deferred as the patient was at elevated risk for the procedure, and was managed conservatively. Trans-thoracic echocardiogram showed moderate calcification of the aortic leaflets; von Willebrand factor (vWF) antigen was 22 IU/dL (normal range 50-200 IU/dL). At the time of discharge, the patient was managed medically on oral estrogen 0.65 mg daily to prevent bleeding from arteriovenous malformations because the cardiac risk stratification put the patient at high risk for any surgical procedure or endoscopic laser treatment.

Despite being on the treatment with oral estrogen patient had recurrent bleeding episodes and received blood transfusions multiple times for recurrent episodes of symptomatic anemia secondary to melena. Unfortunately, the patient passed away because of severe acute on chronic anemia and acute hypoxic respiratory failure secondary to congestive heart failureexacerbation.

## Discussion

The prevalence of Heyde syndrome remains unknown. In one retrospective analysis, 2.6% of patients with aortic stenosis compared with 0.025% of patients in the control group had idiopathic gastrointestinal bleeding [2]. It is postulated that angiodysplasia is a common accompaniment to vascular aging and that bleeding from angiodysplasia lesions in patients with von Willebrand factor deficiency states, therefore, reflects the consequences of a rare hemostatic defect on the backdrop of a common vascular disease [1]. Heyde syndrome appears to consist of bleeding from previously latent intestinal angiodysplasia as a result of this acquired hematological defect, which is associated with aortic stenosis [3].

The cause of the deficiency of high-molecular-weight von Willebrand factor multimers in Heyde syndrome was initially believed to be a consequence of the shear stress-induced binding of von Willebrand factor to platelets, with consequently enhanced clearance [4]. With the identification of shear stress-dependent cleavage of von Willebrand factor in plasma [5], and with the recognition that a plasma protease, ADAMTS13 (a disintegrin and metalloproteinase with a thrombospondin type 1 motif, member 13), is responsible for that cleavage, the pathophysiology of the syndrome became apparent [6]. The acquired reduced levels of large vWF multimers associated with aortic stenosis (causing acquired vWS-2A or von Willebrand syndrome type 2A) lead to impairment of both adhesions and, especially, adenosine 5'- diphosphate-inducibleplatelet aggregation [7], which leads to bleeding from previously latent intestinal angiodysplasia. 

**Figure 2:**
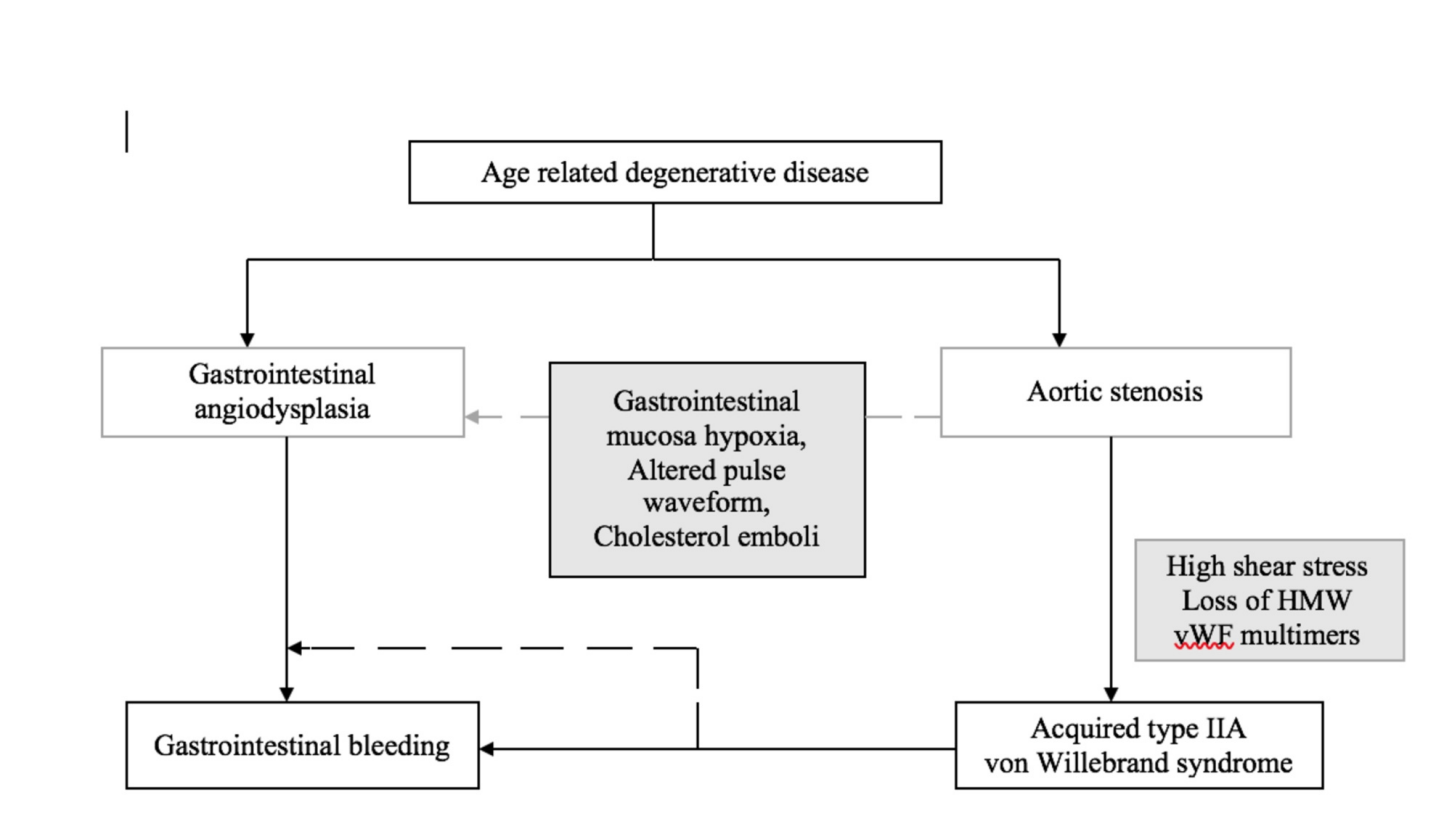
Pathophysiologic mechanisms underlying Heyde syndrome Based on Hudzik et al. [8] HMW: high-molecular-weight; vFW: von Willebrand factor

The pathophysiology is summarized in Figure 2.

Management of Heyde syndrome often requires a multidisciplinary approach, and treatment options include medical therapy, endoscopic interventions, colon surgery, and aortic valve replacement (AVR) [8]. Many elderly patients may be unfit for AVR or may refuse surgery. Conservative management includes oral iron supplements, but regular blood transfusions may be necessary. Combined estrogen and progesterone have been used to reduce bleeding from angiodysplasia, although the mechanism of action is not understood [9]. In serious cases, this could halve transfusion requirements. In patients with severe recurrent bleeding, endoscopy with laser therapy may be an option. In these circumstances, treatment with octreotide may be considered. Many elderly patients have comorbidities requiring anticoagulants or antiplatelet agents, but they should be avoided, particularly in severe cases [10]. 

Aortic valve replacement has been shown to improve hematological abnormalities, and this is paralleled by clinical improvements. Valve replacement appears to offer the best hope of long-term resolution of the bleeding and should be considered in most cases, particularly in those in which theaortic stenosis is symptomatic [3].

## Conclusions

In the elderly population, aortic stenosis is a fairly common finding due to age-related aortic calcification, but patients presenting with concomitant gastrointestinal bleeding should necessitate evaluation for Heyde syndrome. Moderate to severe aortic stenosis has been shown to cause shear-induced von Willebrand factor conformational changes, leading to increased proteolysis of von Willebrand factor, thereby causing deficiency of von Willebrand factor. The deficiency of the von Willebrand factor reduces both adhesion and aggregation of the platelets, thereby causing bleeding from previously latent intestinal angiodysplasia. Association of anemia due to bleeding angiodysplasia, aortic stenosis, and acquired coagulopathy to Heyde syndrome should be high on differentials for these patients as there is a multidisciplinary action plan for these patients, with treatment options ranging from medical treatment with estrogen or thalidomide, etc., to surgical options such as endoscopic laser treatment or aortic valve replacement or colectomy (either partial or total) in severe cases. 
